# Perception of First-Year MBBS Students Toward Virtual Dissection in Learning Anatomy: A Comparative Study Between High and Low Academic Achievers

**DOI:** 10.7759/cureus.72508

**Published:** 2024-10-27

**Authors:** Muralidhar Reddy Sangam, Roonmoni Deka, Vinay G, Praveen K, Amandeep Kaur, Momota Wahengbam

**Affiliations:** 1 Anatomy, All India Institute of Medical Sciences, Guwahati, IND

**Keywords:** academic achievers, educational technology, medical education, perception, virtual anatomy dissection

## Abstract

Introduction

Anatomy, as a crucial subject in the medical curriculum, demands continuous efforts to adopt innovative teaching methods to make it a student-friendly subject. One of the new educational technologies is the virtual anatomy dissection. This high-tech tool allows students to perform some hands-on manipulation of a digital cadaver through an electronic screen in the form of a table. The objectives of the study are to determine the perceptions of first-year MBBS students toward virtual anatomy dissection and to compare the responses of high and low academic achievers.

Methodology

This cross-sectional study was carried out at AIIMS, Guwahati, India, with the approval of the Institutional Ethics Committee (IEC). A validated questionnaire was distributed via Google Forms to 99 students in June 2024. The study used a questionnaire of 20 closed-ended items with a five-point Likert scale and 15 open-ended items to collect data on students’ perceptions toward virtual dissection effectiveness, engagement and interactivity, accessibility, technical usability, learning outcomes, and comparison with traditional dissection. Independent t-test was done to identify statistically significant differences between the responses of high and low academic achievers.

Results

Out of 99 students, 89 (89.89%) responded. Most of the students agreed that virtual dissection is an effective tool for better understanding lectures (65, 73.03%), providing motivation to study (55, 61.79%), making learning a continuous process (57, 64.04%), facilitating deep learning (70, 78.65%), systematic knowledge gain (52, 58.42%), better memorization (72, 80.89%), improved academic performance (63, 70.78%), and reducing anxiety in learning anatomy (42, 47.19%). Sixty-seven (75.28%) stated that virtual dissection should only be a supplement to cadaver dissection for learning Anatomy. Among 20 items (closed-ended questions), only six items showed a statistically significant difference between high and low academic achievers.

Conclusion

The positive feedback from students presents a sound argument that justifies its incorporation into the anatomy curriculum as a supplement for cadaveric dissection.

## Introduction

Traditionally, teaching and learning anatomy has heavily relied on cadaveric dissection, which provides a hands-on, tactile experience that is essential for understanding the complex structures of the human body. As a crucial subject in the medical curriculum, anatomy demands continuous efforts to adopt innovative teaching methods to make it a student-friendly subject. One of the new educational technologies is the virtual anatomy dissection. This high-tech tool allows students to perform some hands-on manipulation of a digital cadaver through an electronic screen in the form of a table. The virtual dissection table allows students to isolate different structures in 3D form, dissect, reconstruct, zoom in and out, and transect them in order to appreciate anatomical form and relationships. Pausing, rewinding, and revisiting different structures and systems by creating presets in a virtual dissection table is a unique feature that helps to provide personalization to the learners [1]. Computer-based simulation materials offer a huge amount of supporting and reinforcing information to learners so that students can work with them at their own pace [2].

The shift toward virtual dissection has sparked considerable debate among educators and students alike. While some students appreciate the flexibility and the ability to learn at their own pace that virtual dissection offers, others express concerns about the lack of tactile feedback and the potential loss of essential skills that come from handling real tissues. Understanding the students’ perceptions of virtual dissection is crucial, as it can inform curriculum design, ensuring that the adoption of new technologies enhances rather than detracts from the educational experience. This understanding can help educators strike a balance between traditional methods and innovative approaches, ultimately improving the efficacy of anatomy education.

Objectives

The objectives are to study the perceptions of first-year MBBS students toward virtual dissection in learning anatomy and to compare the responses of high and low academic achievers toward virtual anatomy dissection.

## Materials and methods

This cross-sectional study was carried out at AIIMS, Guwahati, India, after obtaining permission from the Institutional Ethics Committee (AIIMSG/IEC/M5/F143/2024 dated 31.05.2024). A questionnaire was prepared by the two subject experts based on the findings of a previous study [3] to collect data on students’ perceptions toward virtual dissection effectiveness, engagement and interactivity, accessibility, technical usability, learning outcomes, and comparison with traditional dissection. A pilot study was done with 15 students (selected by convenience sampling) to validate the questionnaire, to test the feasibility of the research design and methods, to check if the students understood all the questions clearly, to make necessary changes to ambiguous questions, and to ensure that the data collected reflects the intended dimensions. The final version of the questionnaire was distributed via Google Forms to the students of first-year MBBS in June 2024. The questionnaire has three parts: the first part included the student demographic data without identity, the second part included 20 items and closed-ended questions with a five-point Likert-scale, which provides a quantitative representation of students’ perception, and the third part included 15 items and open-ended questions, which provide a qualitative representation for deeper understanding of the perceptions. The students were informed about the study details, including confidentiality measures, data protection protocols, the voluntary nature of their participation, and their right to withdraw at any time. Those students who provided their consent were allowed to proceed and complete the questionnaire.

Formative assessments conducted as a part of the regular curriculum for the students in the Department of Anatomy during the academic year were considered to determine academic performance. The students were categorized based on their cumulative academic scores. Thirty students each from the extreme ends of these scores (30 high scorers and 30 low scorers) were grouped as high academic achievers and low academic achievers, respectively. The responses of high and low academic achievers on the Likert scale were compared. For comparative purposes, middle achievers were excluded to capture the clear contrasts in perceptions and derive actionable insights.

Students’ responses on the Likert scale (i.e., strongly disagree, disagree, neutral, agree, and strongly agree) were coded in numerical values (i.e., 1, 2, 3, 4, and 5, respectively) for the purpose of data analysis. Students’ responses to the open-ended questions were read several times, and meaningful units related to students’ perceptions toward virtual anatomy dissection were identified. The meaning units were condensed, interpreted abductively, and finally described as themes by two subject experts. Thematic analysis was conducted by the subject experts to study the key aspects of students’ perceptions: cognitive impact, skill development, emotional response, technical usability, and comparison perspectives.

## Results

Out of 99 students, 89 students gave consent and participated in the study, which is equivalent to a response rate of 89.89%. Sixteen (17.97%) were females, and 73 (82.02%) were males. The responses of the students on the Likert scale were represented as N, percentage, and mean. Independent t-test was done to identify statistically significant differences between the mean Likert scores of high and low academic achievers. A p-value less than 0.05 was considered significant.

**Table 1:** Perception of first-year MBBS students toward virtual dissection in learning anatomy: responses to the closed-ended questions The responses of the students on the Likert scale were represented as N, %, and mean. An independent t-test was done to identify a statistically significant difference between the mean Likert scores of high and low academic achievers. *A p-value less than 0.05 was considered significant. Effect size: Cohen’s d 0.2 (small effect), 0.5 (medium effect), and 0.8 (large effect).

Question	Strongly disagree	Disagree	Neutral	Agree	Strongly agree	Mean Likert score (N = 89)	Mean Likert score among high achievers (N = 30)	Mean Likert score among low achievers (N = 30)	Independent t-test for the significance of the difference between high and low academic achievers’ responses		
	1	2	3	4	5				p-value	Cohen’s d	t-value
I enjoyed learning anatomy through virtual dissection	4 (4.49%)	8 (8.98%)	24 (26.96%)	38 (42.69%)	15 (16.85%)	3.58	4.3	2.8	0.000*	1.6	6.44674
Virtual dissection provides sufficient anatomical knowledge for medical students with no need for lectures	18 (20.22%)	30 (33.7%)	19 (21.34%)	17 (19.1%)	5 (5.61%)	2.56	2.8	2.4	0.101	0.3	1.2873
Virtual dissection helped me understand anatomy lectures in a better way	0	5 (5.61%)	19 (21.34%)	49 (55.05%)	16 (17.97%)	3.85	4.13	3.7	0.018*	0.5	2.13719
Virtual dissection motivates me to study anatomy and attend anatomy lectures	3 (3.37%)	8 (8.98%)	23 (25.84%)	40 44.94%)	15 (16.85%)	3.62	4.03	3.4	0.004*	0.7	2.69165
Virtual dissection makes learning a continuous process	2 (2.24%)	6 (6.74%)	24 (26.96%)	45 (50.56%)	12 (13.48%)	3.66	4.06	3.43	0.001*	0.7	3.06144
Virtual dissection facilitates deep learning of anatomy	1 (1.12%)	2 (2.24%)	16 (17.97%)	48 (53.93%)	22 (24.71%)	3.98	4.2	3.8	0.023*	0.5	2.0354
Knowledge gained through virtual dissection is more systematic	0	7 (7.86%)	30 (33.7%)	35 (39.32%)	17 (19.1%)	3.69	3.76	3.53	0.164	0.2	0.98487
Virtual dissection helped me memorize anatomical details in a better way	0	3 (3.37%)	14 (19.1%)	39 (43.82%)	33 (37.07%)	4.14	4.26	4.1	0.209	0.1	0.81295
Virtual dissection helped me increase my performance in the anatomy exams	1 (1.12%)	4 (4.49%)	21 (23.59%)	45 (50.56%)	18 (20.22%)	3.84	4.03	3.73	0.090	0.3	1.35204
Virtual dissection in groups in the lab is interesting and interactive	2 (2.24%)	3 (3.37%)	14 (15.73%)	52 (58.42%)	18 (20.22%)	3.91	4.1	3.93	0.188	0.2	0.88917
Exposure to virtual dissection reduced my anxiety in learning anatomy and made me comfortable	1 (1.12%)	9 (10.11%)	37 (41.57%)	28 (31.46%)	14 (15.73%)	3.50	3.63	3.5	0.290	0.1	0.5536
I prefer to have an instructor to guide me through virtual dissection	0	6 (6.74%)	12 (13.48%)	44 (49.43%)	27 (30.33%)	4.03	4.06	4	0.386	0.1	0.29035
I prefer to have access to the virtual dissection at any time for self-learning	0	4 (4.49%)	8 (8.98%)	39 (43.82%)	38 (42.69%)	4.24	4.16	4.36	0.180	0.2	-0.9198
I prefer learning anatomy through virtual dissection to cadaver dissection	31 (34.83%)	18 (20.22%)	20 (22.47%)	14 (15.73%)	6 (6.74%)	2.39	2.33	2.4	0.425	0.1	-0.18933
It was difficult to locate and differentiate the structures in the virtual anatomy dissection	9 (10.11%)	22 (24.71%)	35 (39.32%)	18 (20.22%)	5 (5.61%)	2.86	3.23	2.5	0.001*	0.8	3.09425
I do not prefer virtual dissection because of my fear of digital applications	31 (34.83%)	16 (17.97%)	20 (22.47%)	17 (19.1%)	5 (5.61%)	2.42	2.4	2.26	0.347	0.1	0.39513
I recommend using virtual dissection alone without cadaver dissection for learning anatomy	53 (59.55%)	13 (14.6%)	12 (13.48%)	11 (12.35%)	0	1.78	1.86	1.53	0.102	0.3	1.28073
Virtual dissection should only be a supplement to cadaver dissection for learning anatomy	3 (3.37%)	2 (2.24%)	17 (19.1%)	30 (33.7%)	37 (41.57%)	4.07	3.96	4.26	0.152	0.2	-1.03685
I recommend using virtual anatomy dissection for other students and other courses	2 (2.24%)	8 (8.98%)	18 (20.22%)	41 (46.06%)	20 (22.47%)	3.77	3.86	3.76	0.350	0.1	0.38455
Virtual dissection table is essential for medical education	1 (1.12%)	5 (5.61%)	24 (26.96%)	38 (42.69%)	21 (23.59%)	3.82	3.93	3.6	0.091	0.3	1.34967

The responses of first-year MBBS students toward open-ended questions are tabulated in Table 2.

**Table 2:** Perception of first-year MBBS students toward virtual dissection in learning anatomy: responses to the open-ended questions The responses of the students are represented as N and %.

S. no.	Question	Meaningful units (N = 89)	Free text comments
1	Have you had any prior experience with virtual dissection or similar digital anatomy resources before?	21 (23.59%) had a prior experience with virtual dissection.	-
2	How much time per week have you spent learning anatomy using the virtual dissection table?	Meantime is 3.76 ± 2.1 hours (range is 0-10): 4.62 ± 1.5 hours among high achievers and 2.24 ± 0.9 hours among low achievers.	-
3	What would make you use the virtual dissection table more in your self-studies?	Enhanced realism: 68 (76.4%), interactivity: 60 (67.41%), accessibility: 66 (74.15%), and convenience: 52 (58.42%).	“I use virtual dissection as it closely mimics real-life dissection.” “It allows self-paced learning.”
4	What added value does the virtual dissection table have compared to other digital visualization resources like YouTube videos, models, atlas, etc.?	Interactive and immersive learning experience: 56 (62.92%).	“Unlike passive resources like You Tube videos and static models, virtual dissection table allows students to actively explore and manipulate 3D anatomical structures in real-time.” “Hands-on interaction enables deeper understanding of spatial relationships.”
5	How comfortable are you with using technology for learning purposes?	65 (73.03%) are comfortable, and 24 (26.96%) reported initial hesitancy.	“Initially I found it difficult to get the orientation. But as I spend more time, I felt confident in using it.”
6	How do you perceive the level of interactivity?	69 (77.52%) perceived the level of interactivity of virtual anatomy dissection positively, as this hands-on approach enhanced their understanding and retention.	“Virtual dissection table often allow more dynamic exploration of anatomical structures.”
7	Do you think virtual dissection is an effective supplement to traditional cadaver dissection? Why or why not?	67 (75.28%) reported virtual dissection to be an effective supplement to cadaver dissection. The reasons are (a) comprehensive exploration: 29 (32.58%), (b) enhanced understanding: 25 (28.08%), (c) increased engagement: 22 (24.71%), (d) non-availability of cadavers: 20 (22.47%), and (e) no health risk: 12 (13.48%). 66 (74.15%) stated that virtual dissection could not replace cadaver dissection. The reasons are (a) lack of tactile experience: 42 (47.19%), (b) lack of realism and variability: 34 (38.2%), and (c) ethical learning: 18 (20.22%).	“Virtual dissection can be an alternative as where there is a lack of cadavers.” “Interactive features, such as ability to rotate, isolate, and explore structures in 3D, make the study of anatomy more interesting and less monotonous.” “After dissecting virtually, students can reset or reassemble the model for another attempt, which aids in reinforcing learning.” “Students develop surgical skills using surgical tools and understanding the tissue textures, which cannot be replicated with a virtual dissection.” “Handling a human cadaver impart a sense of respect for human body and prepare the students emotionally for the realities of working with patients.”
8	In what ways do you believe virtual dissection can enhance your understanding of three-dimensional anatomy compared to traditional methods?	Interactive learning: 56 (62.92%), repeatability: 50 (53.93%), detailed visualization: 42 (47.19%), customizable learning pace: 32 (35.95%), and dynamic learning: 12 (13.48%).	“Virtual dissection tables provide detailed view of various structures, layer by layer, which can offer a more comprehensive learning experience compared to traditional methods.”
9	What specific features or functionalities of virtual dissection software do you find most helpful in your learning process?	3D rotatable models: 56 (62.92%), layer-by-layer dissection: 42 (47.19%), highlighting and isolating specific structure: 29 (32.58%), adding personal annotations: 18 (20.22%), and cross-sectional views: 9 (10.11%).	-
10	Have you encountered any technical difficulties or limitations while using virtual dissection tools? If so, please describe.	Lack of tactile experience: 68 (76.4%), lack of variations: 12 (13.48%), lack of emotional impact: 11 (12.35%), and dependence on technology, software updates, and internet connectivity: 8 (8.98%).	-
11	How do you feel about the level of detail provided in virtual dissection compared to traditional methods?	Enhanced visualization: 54 (60.67%), interactivity: 36 (40.44%), and consistency: 18 (20.22%).	“Virtual dissection table allows detailed 3D visualization of structures.” “Virtual dissection allows visualization of cross-sections of organs and tissues from various angles which is not possible in traditional dissection.”
12	Do you believe virtual dissection can adequately prepare you for clinical practice? Why or why not?	The response is “yes” by 56 (62.92%) because (a) it provides detailed visualization and special relationships required in the fields of radiology or surgery, and (b) it allows repeated practice to develop competency. 33 (37.07%) responded “no” because of (a) lack of tactile experience and (b) lack of variability.	“Virtual dissection alone may not be sufficient to prepare the student for clinical practice because of lack of tactile experience, which is required for developing the surgical skills, palpation techniques and other hands-on competencies.” “Virtual dissection tables are based on standardized models that may not fully capture the variability found in real human bodies. These variations play a significant role in clinical practice.” “hands-on experience of traditional dissection contributes significantly to their confidence and preparedness for real-world clinical situations.”
13	How do you perceive the role of virtual dissection in fostering collaboration and teamwork among medical students?	It promotes an interactive learning environment: 45 (50.56%); it encourages peer learning and teaching: 24 (26.96%); it enhances communication skills: 13 (14.6%) and interprofessional learning: 5 (5.61%).	“Encourages students to discuss, ask questions and share insights in real-time.” “Peer teaching reinforces the understanding and fosters a supportive learning environment. Diversity of thought enhances learning experience and encourages teamwork.” “It allows medical students to collaborate with students from other health disciplines, fostering teamwork across professional boundaries.”
14	What are the possible challenges faced while learning anatomy through virtual dissection?	Lack of tactile experience: 65 (73.03%), limited realism: 56 (62.92%), reduced emotional impact: 54 (60.67%), cognitive overload: 21 (23.59%), and difficulty in developing clinical skills: 19 (21.34%).	“Virtual dissection table comes with additional information like annotations, layers and interactive features which may be overwhelming and may cause difficulty in focusing on core anatomy learning.” “Virtual dissection leaves the students less prepared for emotional challenges required in clinical practice.” “Variability and complexity of human anatomy is not fully captured in virtual dissection table.” “With virtual dissection it is difficult to understand tissue texture.”
15	What do you think should be improved to make learning through virtual dssection more effective?	By enhancing realism: 69 (77.52%), integrating case-based learning modules: 37 (41.57%), improving real-time collaborative features like shared annotations, group discussions, etc.: 29 (32.58%), intuitive design and user-friendly interface: 17 (19.1%), and incorporating assessment tools: 9 (10.11%).	“Embedding quizzes, self-assessment tools, and immediate feedback mechanisms in the virtual dissection table can help students to monitor their progress." Virtual dissection software must be compatible with wide range of devices like smartphones, low-cost computers, etc.” “Incorporating haptic technology where the students can “feel” the resistance and texture of the tissues.”

## Discussion

Human anatomy, which is the fundamental and basic science in the medical curriculum, is traditionally taught by cadaver dissection and didactic lectures in India. In the present CBME, there is a paradigm shift from passive and teacher-centered learning to active and student-centered learning. CBME is built on key principles such as competency frameworks, integration of theory and practice, assessment and feedback emphasis, individualized learning pathways and flexibility, and promotion of lifelong learning [4]. Though cadaver dissection has proved to be an ideal method of learning anatomy, the increasing number of students per class, the shortage of cadavers, and the need for interactive learning made the stakeholders incorporate information technology in anatomy [5]. These innovations include interactive 3D techniques showing the potential to improve the spatial knowledge of anatomy [6]. Virtual dissection table seemed to be effective for understanding anatomy and knowledge retention, making a favorable impact on students’ learning and perception [1,7]. There are various studies evaluating the integration of technological innovations in gross anatomy [8-16]. The majority of these studies judged virtual dissection as a valuable supplement to traditional teaching methods. There is no clear proof that virtual dissection is preferred by students or could replace cadaver dissection, but there is evidence for its possible role in supplementing traditional teaching methods [17,18]. Many researchers, instructors, and even students believe that employing dissection alone is insufficient to understand anatomy and that adding other educational techniques is advantageous or even needed [19,20].

Funjan et al. studied the perceptions and attitudes of Jordanian medical students on using 3D interactive anatomy dissection in teaching and learning anatomy and reported that the didactic approach that combined cadaver dissection and the virtual dissection table was preferred over the unilateral approach [3]. Ralte et al. assessed the perceived effectiveness of cadaveric, 3D virtual, and combined dissection methodologies and observed a high rating for the combined method [21]. The combination of virtual and traditional gross dissection resulted in a significant improvement in second-year medical students’ learning outcomes. It could help maximize the impact of practical dissection, overcome the contraction of economic resources, and reduce the shortage of available bodies [22].

The 2023 batch of students at our institution experienced learning anatomy by cadaver dissection supplemented by a virtual dissection table during their first professional year. Out of 89, 21 (23.59%) reported having prior experience with virtual dissection or similar digital anatomy resources, and there is no difference between high and low academic achievers. The mean time spent learning anatomy per week using a virtual dissection table is 3.76 ± 2.1 hours. High academic achievers (4.62 ± 1.5 hours) spent more time compared to low achievers (2.24 ± 0.9 hours).

Fifty-three (59.55%) students reported that they enjoyed learning anatomy through virtual dissection, with a statistically significant difference between high achievers (mean score is 4.3) and low achievers (mean score is 2.8). This may be due to initial hesitancy and lack of adequate exposure to digital technologies among low achievers. Fifty-eight (65.16%) students were of the opinion that virtual dissection alone, without the need for lectures, cannot provide sufficient anatomical knowledge.

Sixty-five (73.03%) stated that virtual dissection helps in understanding lectures in a better way, 55 (61.79%) stated that virtual dissection motivates the student to study, 57 (64.04%) stated that virtual dissection makes learning a continuous process, and 70 (78.65%) stated that virtual dissection facilitates deep learning of anatomy. There is a statistically significant difference between high and low academic achievers in these four statements. This may be due to struggle with technical aspects of the software, and virtual dissection requires a certain level of special reasoning and the ability to mentally manipulate 3D objects, which can be challenging to students who are already struggling academically. Low achievers often have lower confidence in their academic abilities. When faced with new and potentially complex tools like virtual dissection, they may feel intimidated, which can foster a negative attitude toward the method.

Majority of the students are of the opinion that virtual dissection helps in systematic knowledge gain (52, 58.42%), better memorization (72, 80.89%), improving academic performance (63, 70.78%), interactive group discussion (70, 78.65%), and reducing anxiety in learning (42, 47.19%). There is no significant difference in these statements between high and low academic achievers. The virtual dissection table provides the same level of access and interaction to all the students, regardless of their prior academic standing. Virtual dissection emphasizes visual and hands-on learning, which may cater equally to different learning styles. Repeated practice and detailed animations may help improve memorization and understanding of the concepts. Some virtual dissection tables come with built-in assessments and instant feedback, allowing students to learn from their mistakes and adapt to their studies in real time.

Seventy-one (79.77%) students preferred to have an instructor to guide through virtual dissection. Seventy-seven (86.51%) preferred to have access to virtual dissection at any time for self-learning. Both high and low academic achievers were of the opinion that instructors can maintain a well-paced learning environment, ensuring the students to stay focused and not lose interest due to a lack of challenge. Instructors can facilitate peer learning and group activities to engage the students in discussions that can potentially improve their performance.

Forty-nine (55.06%) students did not prefer learning anatomy through virtual dissection to cadaver dissection. Sixty-seven (75.28%) students stated that virtual dissection should only be a supplement to cadaver dissection for learning anatomy. High academic achievers who often seek a deep, well-rounded education might view virtual dissection as an excellent tool for initial learning, with cadaver dissection offering the critical hands-on experience that cements their knowledge. Low academic achievers might view virtual dissection as a need for incremental learning, allowing them to learn at their own pace with less pressure. The majority of the students stated that virtual dissection cannot replace cadaver dissection as it lacks realism, variability, and hands-on experience provided by the cadaver dissection.

Apart from various specific added values of virtual dissection, such as comprehensive exploration, interactive learning, repeatability, customizable learning pace, and dynamic learning, a few challenges faced while learning anatomy through virtual dissection, as reported by the students, are lack of tactile experience, limited realism, reduced emotional impact, cognitive overload, and difficulty in developing clinical skills.

The students stated that by enhancing realism, integrating case-based learning modules, improving real-time collaborative features, providing intuitive design and user-friendly interface, and incorporating assessment tools, virtual dissection can be made more effective. Despite the clear advantages of technology-assisted teaching, it is the consensus among educators in the field of anatomy that the role of cadaveric dissection cannot be abolished entirely. Thus, the current trend leans more toward a combined method of teaching that includes both cadaveric dissection and technology-assisted techniques [23].

By thoughtfully blending virtual dissection with traditional methods, medical educators can enhance and create a more flexible, accessible, and engaging learning experience. Selecting the right software and tools, ensuring device compatibility, and establishing a robust support system for troubleshooting software issues can make this integration more successful.

Limitations

The study involves a limited number of students from a single institution, restricting the generalizability of the findings. Further, learning styles and cultural, educational, and curriculum differences may affect the perceptions. As it is a questionnaire-based study, the responses are subjective, capturing the immediate perceptions of the students, and self-reported bias cannot be excluded. As it is an online survey, the other potential limitations are self-selection bias, non-response bias, survey fatigue, distraction, misinterpretation, anonymity, and random responses. Gender-based differences in the perceptions were not studied.

## Conclusions

The present study supports the complementary role of virtual dissection in anatomical education. While it is a powerful tool for initial learning and revision, cadaver dissection remains irreplaceable for mastering the tactile and spatial aspects of anatomy. High academic achievers viewed virtual dissection as an effective tool for deep learning, while low academic achievers took it as a need for incremental learning. The study highlights the need for a balanced approach, integrating both virtual and cadaver dissection to cater to the diverse learning needs of students, ensuring a comprehensive and effective anatomical education. 
